# Genetic analysis of infectious diseases: estimating gene effects for susceptibility and infectivity

**DOI:** 10.1186/s12711-015-0163-z

**Published:** 2015-11-04

**Authors:** Mahlet T. Anche, P. Bijma, Mart C. M. De Jong

**Affiliations:** Animal Breeding and Genomics Centre, Wageningen University, 6700 AH Wageningen, The Netherlands; Quantitative Veterinary Epidemiology Group, Wageningen University, 6700 AH Wageningen, The Netherlands

## Abstract

**Background:**

Genetic selection of livestock against infectious diseases can complement existing interventions to control infectious diseases. Most genetic approaches that aim at reducing disease prevalence assume that individual disease status (infected/not-infected) is solely a function of its susceptibility to a particular pathogen. However, individual infectivity also affects the risk and prevalence of an infection in a population. Variation in susceptibility and infectivity between hosts affects transmission of an infection in the population, which is usually measured by the value of the basic reproduction ratio *R*_*0*_. *R*_*0*_ is an important epidemiological parameter that determines the risk and prevalence of infectious diseases. An individual’s breeding value for *R*_*0*_ is a function of its genes that influence both susceptibility and infectivity. Thus, to estimate the effects of genes on *R*_*0*_, we need to estimate the effects of genes on individual susceptibility and infectivity. To that end, we developed a generalized linear model (GLM) to estimate relative effects of genes for susceptibility and infectivity. A simulation was performed to investigate bias and precision of the estimates, the effect of *R*_*0*_, the size of the effects of genes for susceptibility and infectivity, and relatedness among group mates on bias and precision. We considered two bi-allelic loci that affect, respectively, the individuals’ susceptibility only and individuals’ infectivity only.

**Results:**

A GLM with complementary log–log link function can be used to estimate the relative effects of genes on the individual’s susceptibility and infectivity. The model was developed from an equation that describes the probability of an individual to become infected as a function of its own susceptibility genotype and infectivity genotypes of all its infected group mates. Results show that bias is smaller when *R*_*0*_ ranges approximately from 1.8 to 3.1 and relatedness among group mates is higher. With larger effects, both absolute and relative standard deviations become clearly smaller, but the relative bias remains the same.

**Conclusions:**

We developed a GLM to estimate the relative effect of genes that affect individual susceptibility and infectivity. This model can be used in genome-wide association studies that aim at identifying genes that influence the prevalence of infectious diseases.

## Background

New and existing infectious diseases represent a major and increasing threat to domestic plants and animals, and to humans. Infectious diseases of animals are a worldwide concern, particularly because of their effects on the productivity and welfare of livestock and also because of their zoonotic threats to human health. In spite of the availability of antibiotic and vaccine treatments, the undesirable environmental impact of antibiotic treatments, the rapid evolution of bacteria to develop resistance to antibiotics and of viruses to escape vaccine protection illustrate the need for additional control strategies that can provide a useful complement to the currently used interventions to control disease [1].

Host susceptibility and tolerance are two of the ways that individuals respond to pathogens. Several studies on the genetics of diseases in animals have shown that the host’s susceptibility and tolerance to infectious diseases have a genetic basis, and thus that genotypic differences exist between individuals regarding their susceptibility and tolerance to infectious challenges [2]. A number of genome-wide association studies (GWAS) have reported single nucleotide polymorphisms (SNPs) associated with susceptibility to various infectious diseases [3, 4].

Most genetic approaches that aim at reducing the prevalence of an infection assume that an individual’s disease status (infected/not-infected) is solely a function of its own genes and of non-genetic factors [2]. Hence, these methods capture only the genetic variation in susceptibility or tolerance (strictly, this latter statement is restricted to the measurement of disease occurrence in groups of unrelated individuals [5]). However, the prevalence and dynamics of an infection depend also on the infectivity of infected individuals in the population. Moreover, accumulating evidence on the existence of “superspreaders” in the outbreaks of epidemics suggests that (phenotypic) variation in infectivity exists among hosts [6]. Thus, the classical quantitative genetic approach of disease analysis based on individual disease status will capture only part of the heritable variation that is present in the host population and affects the dynamics of infectious diseases [7].

Between-host variation in susceptibility and infectivity affects the transmission of an infection in the population. This effect is measured by the value of the basic reproduction ratio *R*_*0*_. *R*_*0*_ is defined as the average number of secondary cases produced by one typical infectious individual during its entire infectious lifetime, in an otherwise naïve population [8]. *R*_*0*_ has a threshold value of 1, which implies that a major disease outbreak or a stable endemic equilibrium can only occur when *R*_*0*_ is greater than 1. When *R*_*0*_ is less than 1, the epidemic will die out. Thus, in order to reduce disease incidence and therewith prevalence, breeding strategies should aim at reducing *R*_*0*_, preferably to a value less than 1.

Genetic improvement that aims at reducing *R*_*0*_ should be based on individual breeding values for *R*_*0*_. An individual’s breeding value for *R*_*0*_ is the sum of the average effects of its alleles on *R*_*0*_ [5], which means that investigating the effects of genes on *R*_*0*_ is relevant. Anche et al. [5] showed that an individual’s breeding value for *R*_*0*_ is a function of its genotype for susceptibility and infectivity, and of the population’s average susceptibility and infectivity. Thus, in order to estimate effects of genes on *R*_*0*_, the susceptibility and infectivity effects of the different alleles must be estimated.

Disease data are often available only in binary form (0/1) i.e. the value indicates whether an individual has become infected or not. Hence, methods for genetic analyses of disease traits have to be tailored to such data. Generalized linear models (GLM) are commonly used to analyse binary data, where the expected value of the binary response variable is linked to the explanatory variables (traits) by a linear equation after applying a link function [9]. Velthuis et al. [9] showed that the effect of susceptibility and infectivity of hosts on the transmission rate parameter *β* can be estimated by fitting a GLM with a complementary log–log link function to binary disease data. Lipschutz-Powell et al. [10] showed that a GLM with a complementary log–log link function can be used to link the probability of an individual to be infected to the susceptibility genotype of the individual itself and the infectivity genotypes of its infectious contacts. However, they observed that the infectivity component of the model was non-linear, and did not provide an explicit GLM or investigate the quality of estimates resulting from such a GLM.

In this study, we developed a GLM to estimate the relative effects of genes on individual susceptibility and infectivity, and investigated the quality of the resulting estimates in terms of bias and precision. We also investigated the effect of *R*_*0*_, different sizes of the effects of susceptibility and infectivity genes and population structure with respect to relatedness on bias and precision of the estimates. The GLM was fitted to binary disease data (0/1) recorded at the end of the epidemic. Thus, the data analysed were counts of infected individuals of different genotypes. These data were obtained from a simulated genetically heterogeneous population in which individuals differed in susceptibility and infectivity.

## Methods

### Population structure

We assumed a diploid population with between-host genetic heterogeneity in susceptibility and infectivity. We modelled genetic heterogeneity in this population using two bi-allelic loci, one locus for the susceptibility effect $(\gamma )$ with alleles G and g and susceptibility values $\gamma_{G}$ and $\gamma_{g}$, and one locus for the infectivity effect $(\varphi )$ with alleles F and f and infectivity values $\varphi_{F}$ and $\varphi_{f}$, respectively. Both loci were assumed to have multiplicative allelic effects and the reason for this assumption is explained in the section “Generalized linear models”.

### Epidemiological model of disease dynamics

Disease dynamics that are caused by a microparasitic infection can be modelled with a basic compartmental stochastic susceptible, infected and recovered (SIR) model. In this model, two possible events can occur: infection of a susceptible individual, and recovery of an infectious individual [11]. With stochasticity, these events occur randomly at a certain rate (probability per unit of time) specified by the model parameters and the state variables. In the SIR-model, these parameters are the transmission rate parameter (*β*) for S → I with rate $\beta \frac{SI}{N}$, and the recovery rate parameter (*α*) for I → R with rate $\alpha I$, where *N* denotes population size, *S* the number of susceptible individuals and *I* the number of infectious individuals (in this study, we assumed that an individual will be infectious once it is infected, thus the terms infectious and infected will be used interchangeably; hence, the symbols *S*, *I* and *R* are used to denote both the disease status and the number of individuals with that disease status). The transmission rate parameter *β* describes the probability per unit of time for one infected individual to infect any other individual in a totally susceptible population [8, 12] (this can be seen from the transmission rate $dS/dt = - \beta SI/N$, for *I* = 1 and *S* = *N*).

In the following, we will consider binary data at the end of an epidemic, which indicates for each individual whether it has become infected or not. Thus, binomial count data were available to quantify the occurrence of infected individuals according to genotype. As a step towards the GLM, first we derive the probability of an individual to become infected.

In a genetically heterogeneous population, the transmission rate parameter *β* varies between pairs of individuals, and in addition to the contact rate (c), it will depend on the infectivity genotype of the infectious individual, and on the susceptibility genotype of the recipient susceptible individual. The assumption that the transmission rate depends only on the infectivity of the infectious individual and the susceptibility of the recipient individual, and not on the combination of these two traits, is known as separable mixing [8]. In other words, the two individuals that are in contact influence the transmission rate independently. Thus, the transmission rate of a specific susceptible individual with susceptibility genotype *i* from being susceptible to being infected when exposed to a single infectious individual with infectivity genotype *j* can be defined as:

1 $$\beta_{ij} \frac{1}{N} = \gamma_{i} \varphi_{j} c\frac{1}{N},$$

where $\gamma_{i}$ denotes the susceptibility of the susceptible individual, and $\varphi_{j}$ denotes the infectivity of the infectious individual. Note that the transmission rate in Eq. (1) refers to a single specific susceptible individual, whereas the transmission rate parameter $\beta$ defined above, refers to any susceptible individual among the *N* candidates. Hence, they differ by a factor of *N*. In Eq. (1), *c* represents the average contact rate between any pair of individuals and thus $c/N$ is the average contact rate of a susceptible with a single infectious individual in a group of size *N* (this assumes faecal-oral transmission or similar routes, where $1/N$ of the infectious material ends up with the sender itself). Any variation in contact rate among different types of susceptible and infectious individuals is included in $\gamma_{i}$ and $\varphi_{j}$ because of the assumption of separable mixing.

When one susceptible individual with susceptibility genotype *i* is exposed to one infectious individual with infectivity genotype *j*, the expected number of transmissions is the product of the transmission rate and the average length of the infectious period, and is equal to $\gamma_{i} \varphi_{j} c\frac{1}{N}\frac{1}{\alpha },$ where 1/α is the average length of the infectious period. The probability $P_{ij}$ that the individual escapes infection follows from the zero term of the Poisson distribution, and is equal to:

2a $$P_{ij} = e^{{ - \beta_{ij} \frac{1}{N}}} = e^{{ - \gamma_{i} \varphi_{j} \frac{c}{\alpha }\frac{1}{N}}} .$$

Here, it is assumed that the transmission rate parameter $\beta$ (and thus also $\gamma$, $\varphi$, and $c/\alpha$) is constant over time so that there is no over-dispersion and the Poisson distribution can be used.

At the end of the epidemic, the individual with susceptibility genotype *i* has been exposed not to only one but to all infectious group mates (strictly speaking this is true for the individuals escaping infection only). These group mates can be categorized by their infectivity genotype, *j*. Let *I*_*j*_ denote the number of infected individuals with infectivity genotype *j* that have become infected during the epidemic and have infectivity $\varphi_{j}$. Then the probability $P_{i}$ that the individual escapes all infection exposures by individuals of infectivity genotype *j* and still be susceptible by the end of the epidemic is equal to:

2b $$P_{{i,I_{j} }} = \prod\limits_{{I_{j} }} {e^{{ - \gamma_{i} \varphi_{j} \frac{c}{\alpha }\frac{1}{N}}} } = e^{{ - \gamma_{i} I_{j} \varphi_{j} \frac{c}{\alpha }\frac{1}{N}}} .$$

Thus, the probability $P_{i}$ that the individual with susceptibility genotype *i* escapes all infection exposures from all genotypes and still be susceptible by the end of an epidemic is equal to the product of all the probabilities that it escapes infection exposures from its infectious group mates of each genotype:

3 $$P_{i} = \prod\limits_{j = 1}^{n} {e^{{ - \gamma_{i} I_{j} \varphi_{j} \frac{c}{\alpha }\frac{1}{N}}} } = e^{{ - \gamma_{i} \frac{c}{\alpha }\frac{1}{N}\sum\limits_{j = 1}^{n} {I_{j} } \varphi_{j} }} ,$$

where the summation is over the *n* infectivity genotypes; *n* = 3 for a single bi-allelic locus in a diploid population.

In Eq. (3), we can replace $I_{j}$ by $I \times f_{j}$, where *I* is the total number of individuals that have been infected at the end of the epidemic and $f_{j}$ is the fraction of infected individuals of genotype *j*. This yields:

4 $$P_{i} = e^{{ - \gamma_{i} \frac{c}{\alpha }\frac{I}{N}\sum\limits_{j = 1}^{n} {f_{j} } \varphi_{j} }} .$$

From Eq. (4), the probability that a susceptible individual with susceptibility genotype *i* has been infected by the end of the epidemic is equal to:

5 $$1 - P_{i} = 1 - e^{{ - \gamma_{i} \frac{c}{\alpha }\frac{I}{N}\sum\limits_{j = 1}^{n} {f_{j} } \varphi_{j} }} .$$

Thus, the probability that a susceptible individual has been infected depends on its own susceptibility, $\gamma_{i} ,$ and on the arithmetic mean infectiousness $\sum\nolimits_{j = 1}^{n} {f_{j} } \varphi_{j}$ of its $I$ infectious group mates with different infectivity values $\varphi_{j} ,$ with *j* = 1, … *n*.

In [13], equation 10, which is equivalent to our equation (5), was presented as the final size equation for a population that is heterogeneous for susceptibility and infectivity (in epidemiology, the so-called final size equation gives the fraction of infected individuals of each type by the end of an epidemic). Our equations 5 and 14 in [10] follow a similar derivation but, in our case, the equation is applied to the end of the epidemic.

### Generalized linear model (GLM)

A GLM, in its simplest form, specifies a linear relationship between a function of the mean of the observed variable $y$, and a set of observed predictor variables, $x$:

$$\phi (E(y)) = c_{0} + c_{1} x_{1} + \cdots c_{n} x_{n} ,$$

where $\phi$ is the so-called link function, $c_{0}$ is the intercept and the $c_{i}$ are the regression coefficients for the explanatory variables $x_{i}$, for *i* = 1, … *n*. The aim is to estimate *c*_*i*_ coefficients.

For binomial data where the probability of failure (to escape an infection) P is equal to the zero term of a Poisson distribution, as in the above Eq. (4), the complementary log–log link function is the default link function to connect explanatory variables $x_{i}$ with the observed variable $y$ of the linear model [14]. Applying the complementary log–log link function to $1 - P_{i}$ based on Eq. (4), yields:

6 $${\text{cloglog(}}1 - {\text{P}}_{i} )= {\text{log(}} - {\text{log(}}P_{i} ) )= { \log }\left( {\frac{c}{\alpha }} \right) + \log (\gamma_{i} ) + \log \left( {\frac{I}{N}} \right) + { \log }\mathop \sum \limits_{j = 1}^{n} f_{j} \varphi_{j}$$

Thus, the dependent variables have now become the fraction of each *i* type of individual that did become infected (see below).

The model in Eq. (6) is linear in log of susceptibility $(\gamma_{i} )$ but not for infectivity $(\varphi_{j} )$, since the logarithm of a sum does not equal the sum of the logarithms, as also observed by [11]. In Eq. (6), the term $\sum\nolimits_{j = 1}^{n} {f_{j} } \varphi_{j}$ can be recognized as the arithmetic mean, since $\sum\nolimits_{j = 1}^{n} {f_{j} } = 1$. In order to further linearize Eq. (6), the arithmetic mean was approximated by a geometric mean, using the substitution $\sum\nolimits_{j = 1}^{n} {f_{j} } \varphi_{j} \approx \prod\nolimits_{j = 1}^{n} {\varphi_{j}^{{f_{j} }} }$. This yields:

7 $$\log ( - {\text{log(}}P_{i} )) \approx\;\log \left( {\frac{c}{\alpha }} \right) + \log (\gamma_{i} ) + \log \left( {\frac{I}{N}} \right) + \mathop \sum \limits_{j = 1}^{n} f_{j} {\text{log}}(\varphi_{j} ) .$$

The approximation of the arithmetic mean regression by a geometric mean regression was investigated separately as explained in the “Appendix”.

In Eq. (7), the expectation of the response variable, $cloglog(1 - P_{i} )$ is a linear expression of ${ \log }(\gamma_{i} )$ and ${ \log }(\varphi_{j} )$.

Equation (7) is linear in the log of susceptibility $(\gamma_{i} )$ and the log of infectivity $(\varphi_{j} )$. To be able to formulate the model in terms of allele counts within individuals, rather than in terms of individual genotypes, it was assumed that the two alleles that make up the genotype within an individual act multiplicatively, so that their effects are additive on the log-scale.

Therefore, the genotypic values will be $\gamma_{GG} = \gamma_{G} \times \gamma_{G} = \gamma_{G}^{2}$, $\gamma_{gg} = \gamma_{g} \times \gamma_{g} = \gamma_{g}^{2}$ and $\gamma_{Gg} = \gamma_{gG} = \gamma_{G} \times \gamma_{g}$, for susceptibility, and $\varphi_{FF} = \varphi_{F} \times \varphi_{F} = \varphi_{F}^{2}$; $\varphi_{ff} = \varphi_{f} \times \varphi_{f} = \varphi_{f}^{2}$ and $\varphi_{fF} = \varphi_{Ff} = \varphi_{f} \times \varphi_{F}$ for infectivity. Furthermore, the effects of the $g$ and $f$ alleles were set to a value of 1, $\gamma_{g} = 1 = \varphi_{f} = 1$, so that ${ \log }(\gamma_{g} ) = { \log }(\varphi_{f} ) = 0$. This is done without loss of generality, because the interest lies in the relative effect of one allele to the other, that is the effect of $\gamma_{G}$ relative to $\gamma_{g}$ and the effect of $\varphi_{F}$ relative to $\varphi_{f}$ [note that this does not affect the estimates of relative allele effects since the absolute scale of the model is accounted for by the log(c/α)-term]. Using Eq. (7), the GLM for the diploid genetic model becomes:

8 $${\text{cloglog}} E\left[ {\frac{{y_{i} }}{{n_{i} }}} \right] = c_{0} + c_{1} index_{G,i} + c_{2} Num_{F} + log\left( {\frac{I}{N}} \right),$$

where individuals are aggregated by their genotype, *i*. The ${\text{cloglog}}$ is applied to the expectation of $\frac{{y_{i} }}{{n_{i} }}$, which is the fraction of infected individuals of genotype *i*, by the end of the epidemic and $y_{i}$ follows a binomial distribution, $c_{0}$ is the intercept measuring log(c/α), and $c_{1}$ is the regression coefficient for the $index_{G}$, where $index_{G,i} = 0,\;1\;{\text{or}}\;2$ is the number of G alleles at the susceptibility locus of individuals of genotype $i$. The $c_{2}$ is the regression coefficient for $Num_{F}$, which is the average of the number of F-alleles per individual at the infectivity locus in the infected group mates of the individuals of genotype *i*. It is calculated as $2 \times Frac_{FF} + 1 \times Frac_{fF/Ff}$ where $Frac_{FF}$ is the fraction of infected individuals with genotype $\hbox{''}FF\hbox{''}$ and $Frac_{fF/Ff}$ is the fraction of infected individuals with genotype “*fF*” *or* “*Ff*”. The “2” arises because individuals with the $\hbox{''}FF\hbox{''}$ genotype carry two F alleles, while those with the “*fF*” *or* “*Ff*” genotype carry only one F allele. The ${ \log }\left( {\frac{I}{N}} \right)$ corresponds to the total fraction of infected individuals in the group, which is used as an offset in the GLM. Hence, estimates of *c*_1_ and *c*_2_ refer to the effect of a single allele, and represent the so-called average effect of an allele substitution on the log-scale [15]. When fitting the model to binomial count data of those individuals of each genotype that are infected and estimating *c*_0_, *c*_1_ and *c*_2_, the effects of alleles G and F relative to $\gamma_{g}$ = $\varphi_{f}$ = 1 can be calculated as $\widehat{\gamma }_{G} = e^{{\widehat{{c_{1} }}}}$ and $\widehat{\varphi }_{F} = e^{{\widehat{{c_{2} }}}}$, respectively.

### Simulation

To investigate the bias and precision of the $\widehat{\gamma }_{G}$ and $\widehat{\varphi }_{F}$, one generation of a diploid population was simulated based on the above assumptions with respect to the effect of alleles at both loci. These two loci were the only genetic effects simulated. Furthermore, it was assumed that allele frequencies at both loci were equal to 0.5, that is, $p_{g} = p_{f} = 0.5$. The population was sub-divided into 100 groups of 100 individuals each. Each group was set up in such a way that group mates showed a certain genetic relatedness, *r*, at both loci. Here, relatedness is defined as the correlation of allele counts between group mates, irrespective of what causes the correlation. To limit the number of scenarios to be tested, relatedness at the susceptibility locus, $r_{\gamma }$, and at the infectivity locus, $r_{\varphi } ,$ were assumed to be the same (note that relatedness at both loci is expected to be the same when the loci are not under selection). In order to have a certain degree of relatedness among group mates, a fraction of fully related individuals was added to each group, supplemented by randomly selected individuals. Since each individual carries both the susceptibility and the infectivity locus, these additions were done jointly (see Appendix 4 in [5] for a detailed description of the strategy to make these additions jointly).

A basic stochastic SIR-model as described above was used to simulate the disease dynamics [12]. In each group, the epidemic began by one randomly infected individual. Then, the next event which could be either infection of a susceptible individual or recovery of infected individual was determined using Gillespie’s direct algorithm [16]. The type of event, i.e. either infection or recovery, was decided by drawing a random number *v*_*1*_, from a uniform distribution, *v*_*1*_ ~ U(0,1). The next event was an infection of a susceptible individual if the random number $v_{1} < \frac{{\mathop \sum \nolimits_{i} \mathop \sum \nolimits_{j} \beta_{ij} \frac{{S_{i} I_{j} }}{N}}}{{\mathop \sum \nolimits_{i} \mathop \sum \nolimits_{j} \beta_{ij} \frac{{S_{i} I_{j} }}{N} + I\alpha }},$ otherwise it was recovery of an infected individual. The numerator of this ratio represents the total infection rate, and the denominator the total rate, i.e., the sum of the infection and recovery rates. The sampling of the specific individual that became infected depended on individual susceptibility. The probability that a susceptible individual of genotype *i* became infected was proportional to $\frac{{\mathop \sum \nolimits_{i} \mathop \sum \nolimits_{j} \beta_{ij} \frac{{S_{i} I_{j} }}{N}}}{{\mathop \sum \nolimits_{i} \mathop \sum \nolimits_{j} \beta_{ij} \frac{{S_{i} I_{j} }}{N} + I\alpha }}$. Hence, the transmission rates were updated based on the numbers of susceptible and infected individuals of each genotype, while the transmission rate parameter $\beta_{ij}$ remained constant. The epidemic ended when there was no more infectious individual in the population or when there was no susceptible individual left to be infected. By the end of the epidemic, the number of individuals that got infected together with their genotypes for susceptibility and infectivity were recorded. The fraction of individuals of each genotype that got infected was the dependent variable in the analysis.

We hypothesised that different epidemiological and genetic factors will affect the quality of the estimates, as measured by the bias and precision of $\widehat{\gamma }_{G}$ and $\widehat{\varphi }_{F}$. For that purpose, we simulated different scenarios that are described below. The biases of the estimates were calculated by taking the difference between the ‘true’ and estimated values and the precision of the estimates were calculated using the standard deviation (SD) of the estimates.

First, we simulated a basic scenario (scenario 1; Table 1), in which groups were created randomly with respect to relatedness among group mates. We calculated *R*_*0*_ using [5]:

$$R_{0} = \overline{\gamma } \overline{\varphi } {c}/{\alpha},$$

where

$$\overline{\gamma } = p_{g}^{2} \gamma_{gg} + 2p_{g} (1 - p_{g} )\gamma_{gG} + (1 - p_{g} )^{2} \gamma_{GG} ,$$

and

$$\overline{\varphi } = p_{f}^{2} \varphi_{ff} + 2p_{f} (1 - p_{f} )\varphi_{fF} + (1 - p_{f} )^{2} \varphi_{FF} .$$

Population parameters are in Table 1. In the basic scenario, *R*_*0*_ was set to 1.2.

**Table 1:** Simulated scenarios

Parameters	Scenario 1	Scenario 2	Scenario 3
Contact rate, *c*	1.5	0.75–7.5	1.5
Recovery rate *α*	0.5	0.5	0.5
$\gamma_{G}$	0.6	0.6	0.97, 0.6 and 0.37
$\varphi_{F}$	0.6	0.6	0.3, 0.6 and 0.9
Relatedness *r*	0–1	0–1	0–1
*R*_*0*_	1.2	0.6–6.1	1.2

For all scenarios, $\gamma_{g} = \varphi_{f} = 1$ and $p_{g} = p_{f} = 0.5$

Second, to investigate the effect of *R*_*0*_ on the quality of $\widehat{\gamma }_{G}$ and $\widehat{\varphi }_{F}$, we simulated scenarios with different values of *R*_*0*_. We varied the contact rate *c*, so that *R*_*0*_ for a population consisting of groups with unrelated individuals varied from 0.6 (for which no major outbreaks can occur) to 6.1 (for which major outbreaks can occur; Table 1, scenario 2).

Third, to investigate the impact of the size of effects of the genes for susceptibility and infectivity on the quality of $\widehat{\gamma }_{G}$ and $\widehat{\varphi }_{F}$, we simulated scenarios with different effect sizes for a constant value of *R*_*0*_ = 1.2. We simulated all combinations of low, moderate and high values for $\gamma_{G}$ and $\varphi_{F}$ (Table 1, scenario 3).

Furthermore, in all of the above-mentioned scenarios, relatedness between group mates was varied between 0 and 1 to investigate the effect of population structure with respect to relatedness on the quality of $\widehat{\gamma }_{G}$ and $\widehat{\varphi }_{F}$. Relatedness was assumed to be the same at both loci (see [5] for details). We used R software to fit the model with a *glm* function and a binomial distribution.

## Results

**Fig. 1:**
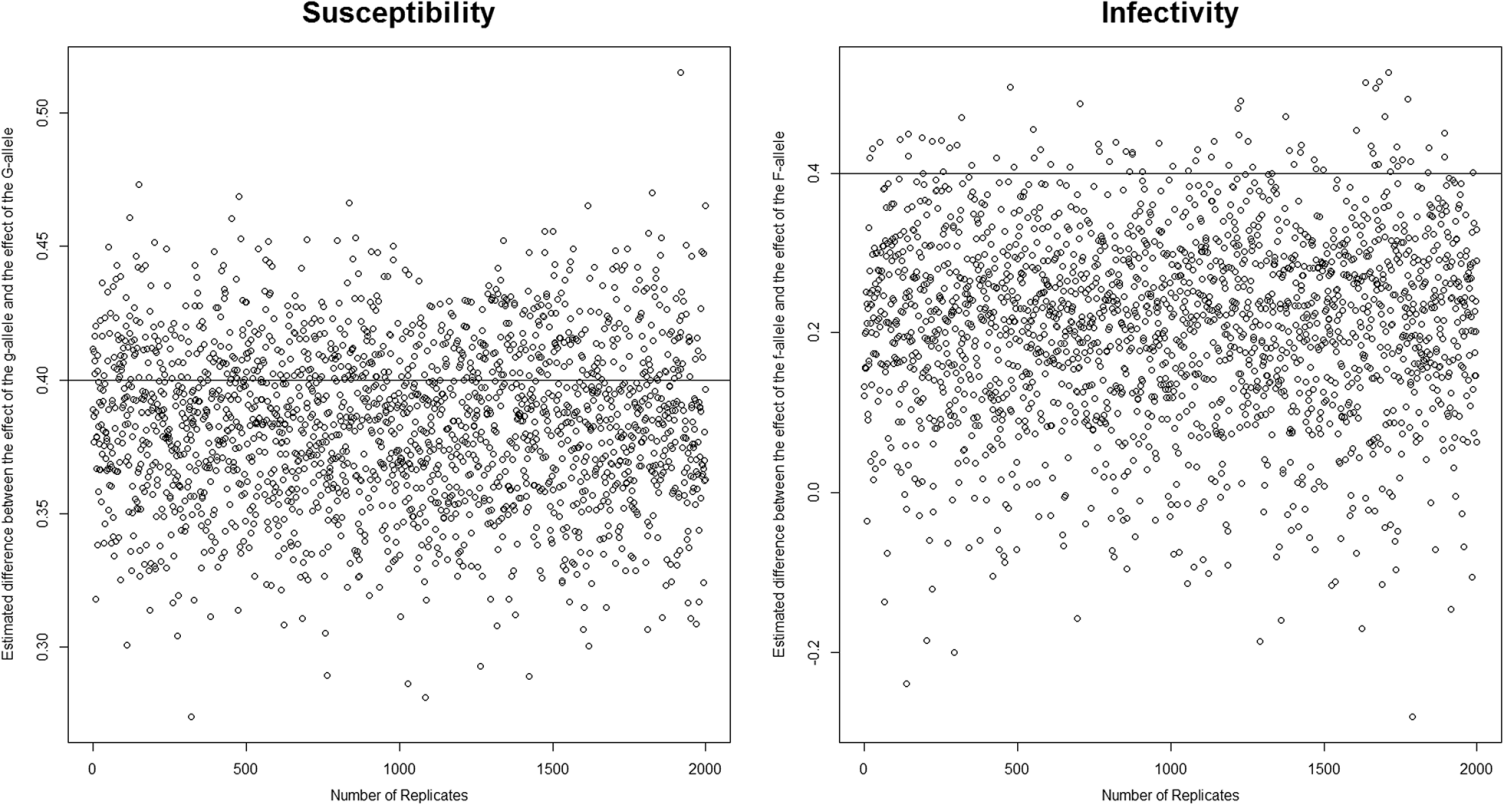
Scatter plots for $(1 - \widehat{\gamma }_{G} )$ and infectivity $(1 - \widehat{\varphi }_{F} )$. For the scenario where relatedness between group mates *r* = 0 and *R*_*0*_ = 1.2

All estimates presented in this section are averages from 2000 replicates, except for Fig. 1 which shows the results of all replicates. The black straight line in all figures represents the true difference between $\gamma_{g}$ and $\gamma_{G}$ and between $\varphi_{f}$ and $\varphi_{F}$, and the bars indicate the standard deviation of these estimates among replicates.

**Fig. 2:**
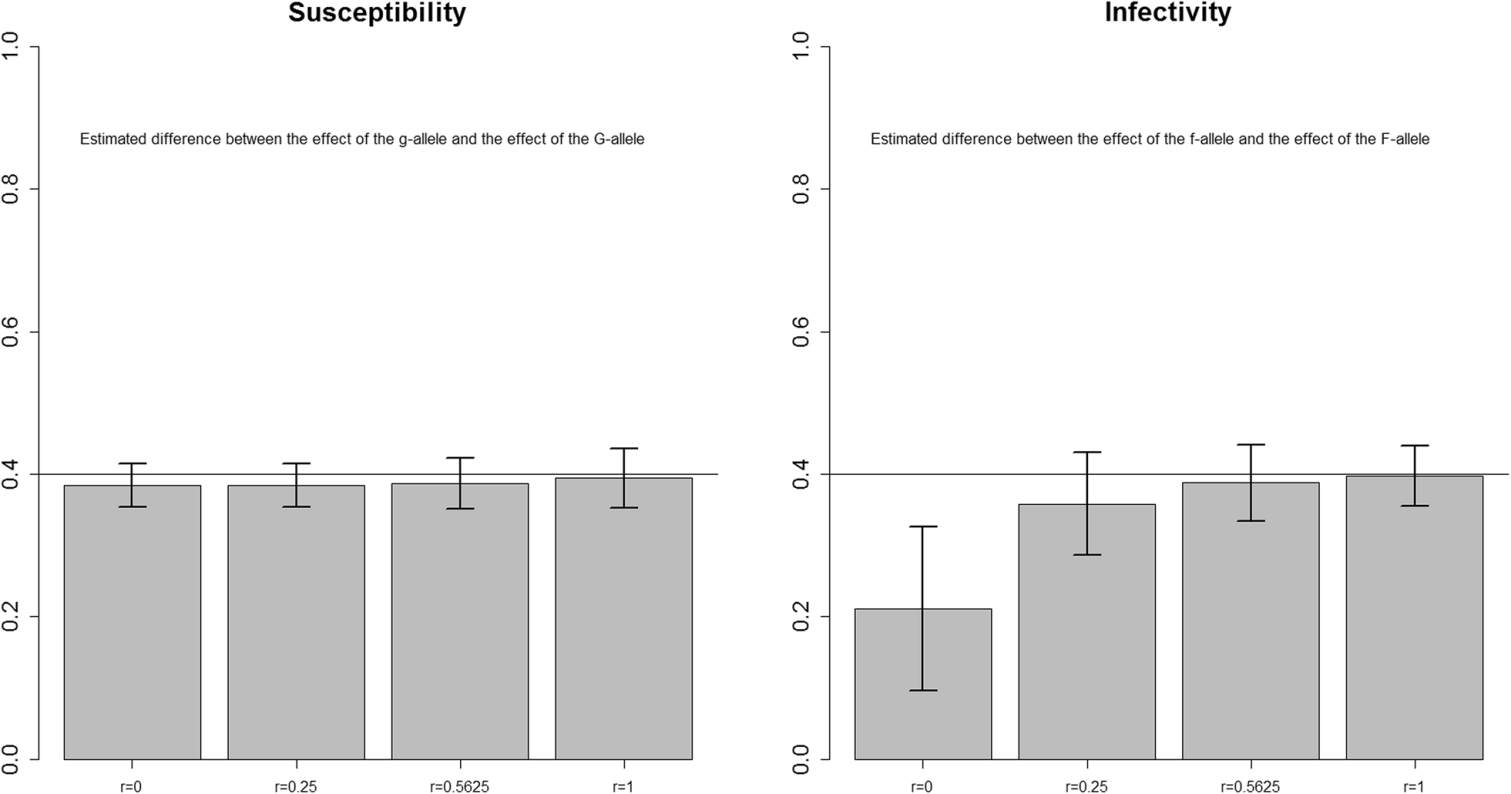
Difference between $\gamma_{g}$ and $\widehat{\gamma }_{G}$ and between $\varphi_{f}$ and $\widehat{\varphi }_{F}$. For the scenario with different values of relatedness *r* between group mates, and $\gamma_{g} = \varphi_{f} = 1$

In the basic scenario, in which groups were created randomly with respect to relatedness, *r* = 0, we found that the susceptibility effect was slightly underestimated ($1 - \widehat{\gamma }_{G}$ in Fig. 2) but the infectivity effect was considerably overestimated ($1 - \widehat{\varphi }_{F}$ in Fig. 2). When the degree of relatedness among group mates increased, the bias of both estimates decreased, however, the effect of relatedness was more pronounced for infectivity (Fig. 2). The error in $\widehat{\varphi }_{F}$, that is caused by the geometric mean approximation was quantified and found to be small (Table 3, “Appendix”). Moreover, the standard deviation of the estimated susceptibility effect increased only slightly, whereas the standard deviation of the estimated infectivity effect increased considerably as the degree of relatedness increased.

A scatter plot for $(1 - \widehat{\gamma }_{G} )$ and $(1 - \widehat{\varphi }_{F} )$ of the 2000 replicates for the basic scenario where *r* = 0 shows that the estimated differences are uniformly distributed over their range without any pattern (Fig. 1). This plot also shows that $(1 - \widehat{\varphi }_{F} )$ is more often underestimated than overestimated, which agrees with the underestimation in Fig. 2 for *r* = 0.

**Fig. 3:**
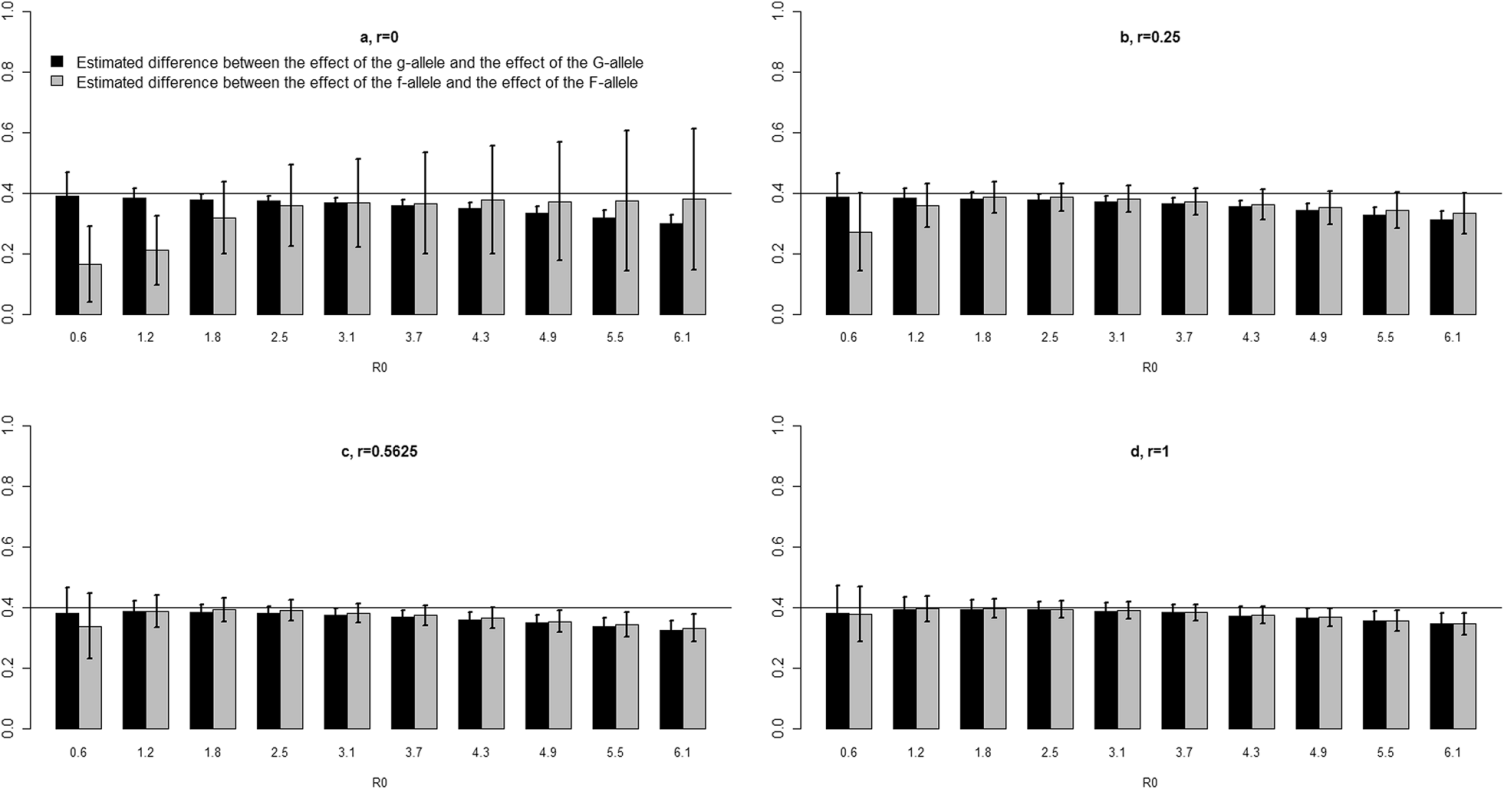
Difference between $\gamma_{g}$ and $\widehat{\gamma }_{G}$, and between $\varphi_{f}$ and $\widehat{\varphi }_{F}$. For the scenario with different values of *R*_*0*_ and degrees of relatedness *r* between group mates. ($\gamma_{g} = \varphi_{f} = 1)$

In the second set of scenarios, where *R*_*0*_ was varied from 0.6 to 6.1, susceptibility and infectivity effects were also underestimated. Bias in $\widehat{\gamma }_{G}$ and $\widehat{\varphi }_{F}$ was smallest for values of *R*_*0*_ that ranged approximately from 1.8 to 3.1. Higher values of *R*_*0*_ increased bias in $\widehat{\gamma }_{G}$ but had little effect on bias in $\widehat{\varphi }_{F}$ when group mates were unrelated (Fig. 3, panel a). Bias in $\widehat{\varphi }_{F}$ and $\widehat{\gamma }_{G}$ decreased with increasing relatedness among group mates, except for $\widehat{\varphi }_{F}$ at high values of *R*_*0*_ (Fig. 3, panels b–d). In contrast to the result for the unrelated groups, bias in $\widehat{\varphi }_{F}$ was larger at high values of *R*_*0*_ when related groups were used (Fig. 3, panel a vs. b–d). For fully-related groups, i.e. *r* = 1, estimates for $\widehat{\varphi }_{F}$ and $\widehat{\gamma }_{G}$ and their standard deviation were nearly identical (Fig. 3, panel d). For this scenario, the error in $\widehat{\varphi }_{F}$ as a result of the geometric mean approximation was also quantified and only a small error was found (“Appendix”, Table 4).

For all values of *R*_*0*_, standard deviations of estimates were greater for infectivity effect than for susceptibility effect, except for *r* = 1 for which they were nearly identical. Standard deviations decreased considerably as relatedness among group mates increased, particularly for infectivity effect. For both susceptibility and infectivity effects, standard deviations were smaller for values of *R*_*0*_ for which the bias in $\widehat{\gamma }_{G}$ and $\widehat{\varphi }_{F}$ was smallest, i.e. when *R*_*0*_ ranged approximately from 1.8 to 3.1.

**Fig. 4:**
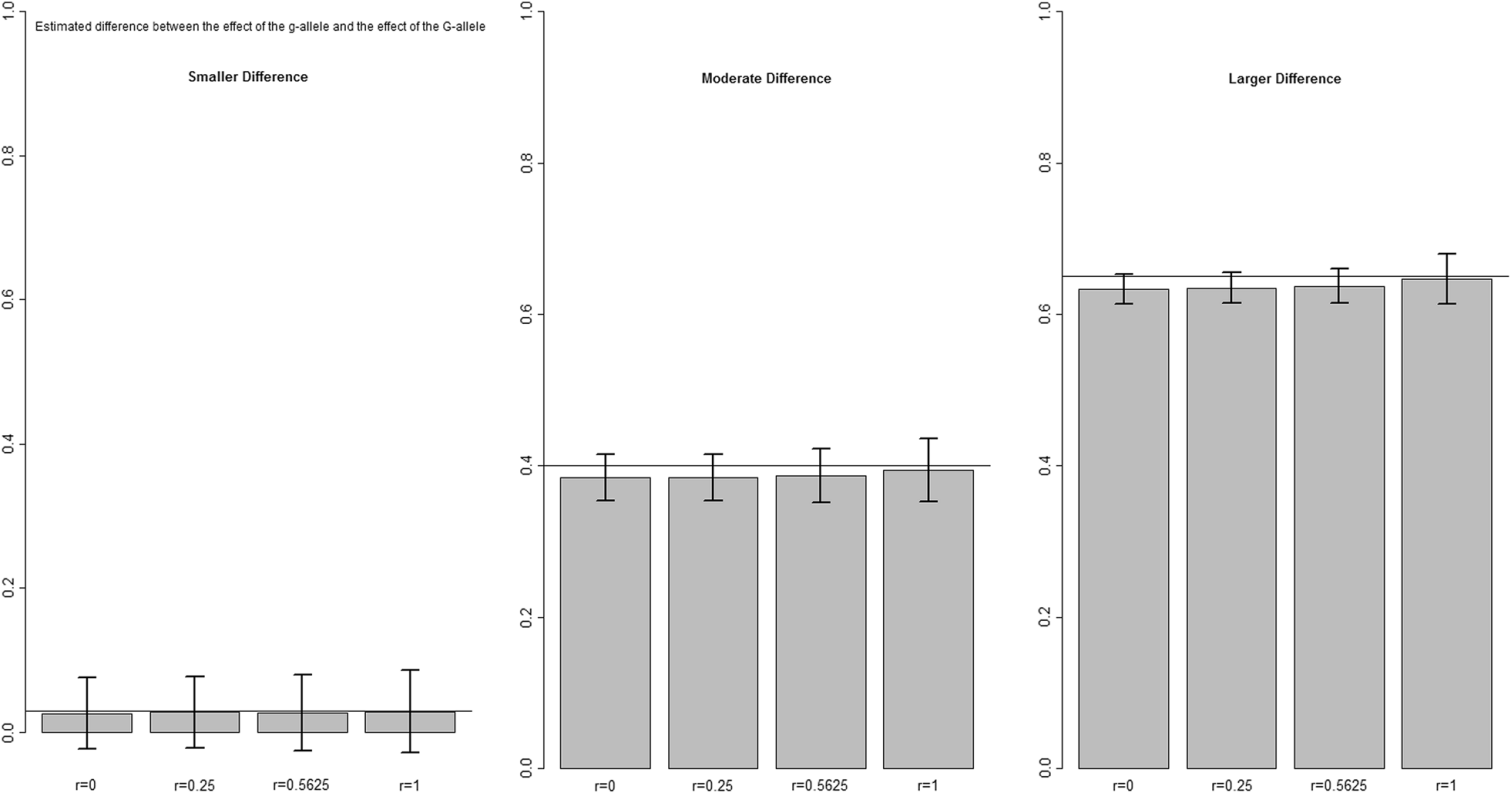
Difference between $\gamma_{g}$ and $\widehat{\gamma }_{G}$. For the scenario with different values of the (true) difference between $\gamma_{g}$ and $\gamma_{G}$, and for different values of relatedness *r* between group mates, and *R*_*0*_ = 1.2. ($\gamma_{g} = \varphi_{f} = 1)$

**Fig. 5:**
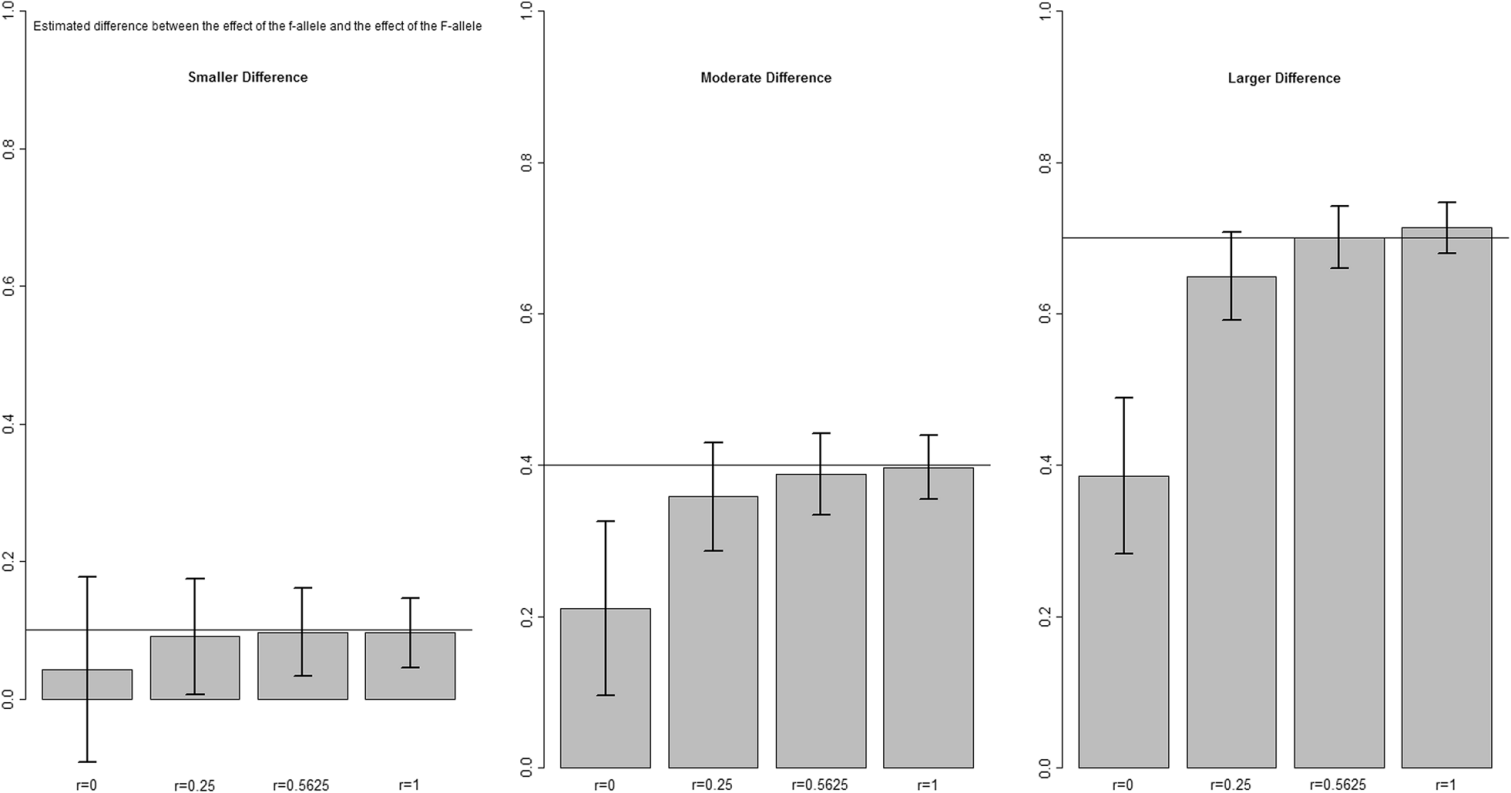
Difference between $\varphi_{f}$ and $\widehat{\varphi }_{F}$. For the scenario with different values of the (true) difference between $\varphi_{f}$ and $\varphi_{F}$, and for different values of relatedness *r* between group mates and *R*_*0*_ = 1.2. $(\gamma_{g} = \varphi_{f} = 1)$

In the third set of scenarios, different sizes of the effects of $\gamma_{G}$ and $\varphi_{F}$ were simulated. For both estimates, the relative bias did not change regardless of the size of the effect considered (Figs. 4, 5). In these scenarios also, both susceptibility and infectivity effects were underestimated regardless of the size of the effects considered, except when there was a large difference in infectivity effect and *r* = 1, there was a small overestimation ($1 - \widehat{\varphi }_{F}$ in Fig. 5). Moreover, smaller relative standard deviations were found for both susceptibility and infectivity effects when effect sizes were larger, which indicates that the effects are better estimated when they are larger. For this scenario, the error in $\widehat{\varphi }_{F}$ as a result of the geometric mean approximation was also quantified and only a small error was found (Table 5, “Appendix”).

## Discussion

In this work, a generalized linear model with a complementary log–log link function was developed to estimate the relative effects of genes on individual susceptibility and infectivity. The model was developed from an equation that describes the probability of an individual to become infected as a function of its own susceptibility genotype and of the infectivity genotypes of its infected group mates. This GLM was developed following Velthuis et al. [9] who developed a GLM for binary data on a transmission trial to estimate the effect of susceptibility and infectivity of hosts on the transmission rate parameter *β.* A simulation study was performed to investigate the quality of the GLM. From the statistical analysis of the simulated data, we obtained fairly precise estimates, except for some scenarios for which estimates were more biased, particularly for infectivity. The best estimates were found for schemes with intermediate *R*_*0*_ and related group members. For all the scenarios investigated, the sizes of the effects at both loci were underestimated.

The main objective of this study was to develop a methodology to estimate gene effects and also to investigate its quality in terms of bias and precision of the estimates. To test the methodology without introducing additional assumptions that may contribute to estimation error, we assumed additive allele effects on the log-scale for both susceptibility and infectivity. Thus, allelic effects were simulated multiplicatively on the original scale. This was done for two reasons. First, we wanted to formulate the model in terms of allele counts within individuals, rather than in terms of individual genotypes. In other words, we did not intend to estimate dominance effects. Whether allele effects are more likely to be additive on the log-scale than on the original scale is unknown at present. Second, since the objective of this study was to investigate the quality of the model rather than the assumptions on the genetic architecture, the data were simulated under a model that agreed with the assumptions of the statistical model.

Bias and standard deviation of the estimates were smallest for *R*_*0*_ that ranged approximately from 1.8 to 3.1. The basic reproduction ratio *R*_*0*_ is an important factor that affects the size of an epidemic in a population, i.e. the fraction of individuals that are found to be infected by the end of an epidemic. When *R*_*0*_ is greater than 1 but near 1 in a group, there will be virtually no individuals infected and thus, there is hardly any variation in disease status, which results in inaccurate estimates of gene effects. Conversely, when *R*_*0*_ is much greater than 1, nearly all individuals will be infected, which again results in very little variation in disease status. (Table 2 indicates the fraction of infected individual for different values of *R*_*0*_ and relatedness among group mates). Thus, the relationship between *R*_*0*_ and the fraction of individuals infected affects the estimation of the effect on susceptibility and infectivity_,_ since data on the final size of an epidemic were used for our estimation. This mechanism may explain why the estimated effect on susceptibility is best for intermediate *R*_*0*_. The effect of infectivity is more difficult to estimate and the bias is larger.

**Table 2:** Fraction of individuals infected at the end of the epidemic

	*r* = 0	*r* = 0.25	*r* = 0.5625	*r* = 1
*R*_*0*_ = 0.6	0.02	0.03	0.03	0.04
*R*_*0*_ = 1.2	0.10	0.12	0.14	0.16
*R*_*0*_ = 1.8	0.30	0.30	0.30	0.30
*R*_*0*_ = 2.5	0.46	0.45	0.44	0.43
*R*_*0*_ = 3.1	0.58	0.57	0.55	0.53
*R*_*0*_ = 3.7	0.66	0.65	0.63	0.61
*R*_*0*_ = 4.3	0.71	0.70	0.69	0.67
*R*_*0*_ = 4.9	0.75	0.75	0.73	0.71
*R*_*0*_ = 5.5	0.79	0.78	0.77	0.75
*R*_*0*_ = 6.1	0.81	0.80	0.80	0.78

For each scenario, more relatedness between individuals resulted in better estimates for both traits. This is because more relatedness creates more variation between groups, which results in groups with below or above average susceptibility and/or infectivity. This occurs because an individual with a lower susceptibility will also have related group mates with below average susceptibility, and vice versa. The same applies for infectivity. However, since we assumed absence of linkage disequilibrium (LD) between the susceptibility and infectivity loci, groups with below average susceptibility will not always have below average infectivity as well. Thus, only those groups with above average susceptibility and above average infectivity will have epidemics with a greater final size, i.e. the fraction of individuals that gets infected by the end of the epidemic, while those with below average susceptibility and infectivity will have a lower final size. This variation improved estimates of the effects of susceptibility and infectivity.

We have made a number of assumptions in building our methodology. In the derivation of Eq. (5), we assumed that all individuals that escaped the infection had been exposed to all infected individuals. Of course, this assumption is true for the simulations done here. To what extent, this will be true for real data remains to be seen. It seems reasonable to assume that individuals in relatively small and well-defined groups get mixed up over space and time as is often the case in animal husbandry: for example, in fattening pigs with group sizes of 10 to 30 individuals. The assumption is less reasonable for groups with a spatial structure, for example in tie stalls or when epidemics occur within barns subdivided into multiple groups. In such cases, data should be collected separately for different groups. We also assumed that epidemics could be completely recorded, so that the final disease status of all individuals is known, and all individuals that have escaped the infection have been exposed to all infected individuals. However, for reasons of, e.g., animal welfare and productivity, interventions are often carried out to limit the size of an epidemic. Hence, individuals may not have had the full potential to express their susceptibility and infectivity. For incomplete epidemics, the probability that an individual becomes infected follows from Eq. (5) when only the infected individuals to which the focal individual has been exposed are considered (see also [11]). Thus, extension to incompletely observed epidemics is straightforward (see also application in [_ENREF_189] and subsequent papers citing [9]).

Bias and precision of estimates may be improved when data are recorded within shorter time intervals. This may be particularly helpful for cases with high *R*_*0*_. In such cases, each interval forms an incompletely observed epidemic, which can be analysed with the same GLM statistical approach [9]. When data are collected in sufficiently short time intervals, only a fraction of individuals will become infected in a single interval, even when *R*_0_ is high. This will contribute to accuracy of the estimates. Moreover, collecting data in short time intervals also provide information on the order of infections, i.e., which animal has infected which animal. This will increase the accuracy of estimated gene effects, particularly for infectivity [17]. Thus, using data from short time intervals can be complementary to using groups composed of related individuals and data from multiple epidemics. The derivation and resulting model for such cases is very similar to the one presented here, since the probability that an individual escapes infection follows from the zero-term of the Poisson distribution (see also [9, 11]). The key step is to identify the infectious individuals to which the focal individual has been exposed in a time period.

Lipschutz-Powell et al. [11] showed that, when there is genetic variation in susceptibility only, a complementary log–log link function can be used to link an equation that describes the probability of an individual to become infected to a linear model that includes the individual’s genotype for susceptibility. They also suggested that, when there is genetic variation in infectivity, a Taylor-series expansion of the model term for infectivity can be used to further linearize the model in infectivity. In our study, we obtained a linear model for infectivity by approximating the arithmetic mean by a geometric mean. We quantified the error due to this approximation and found only negligible errors in the estimates (“Appendix”). Thus, this approximation can be ruled out as the cause of the observed bias. This suggests that, for cases for which there is variation in infectivity, the geometric mean approximation is suitable to obtain a linear combination of the parameters of interest. A full investigation of the causes of the bias is beyond the scope of this study. However, the fact that a population of finite size, i.e., 100 individuals in each group, was used to estimate gene effects can be one of the reasons for the observed underestimation.

Anche et al. [5] defined breeding value and heritable variation in *R*_*0*_. They showed that an individual’s breeding value for *R*_*0*_ is a function of the population’s average susceptibility and infectivity, of the gene frequencies within the individual and of average effects of the alleles at both loci (Equation 7c in [5]). However, Anche et al. [5] assumed that effects of alleles at both loci were additive, whereas here we assumed that effects are multiplicative (so that they are additive on the log scale). Multiplicative effects introduce dominance. Hence, before applying the expressions for breeding value and heritable variation of [5] to estimates obtained from the methods proposed here, they need to be translated into average effects of alleles [15]. Using the common notation for the one-locus model [15], the additive effect is half the difference in genotypic value between both homogyzotes, $a_{\gamma } = (\gamma_{g}^{2} - \gamma_{G}^{2} )/2$ and $a_{\phi } = (\phi_{f}^{2} - \phi_{F}^{2} )/2$, the dominance deviation is the difference between the heterozygote and the average of both homozygotes, $d_{\gamma } = \gamma_{g} \gamma_{G} - (\gamma_{g}^{2} + \gamma_{G}^{2} )/2$ and $d_{\varphi } = \varphi_{f} \varphi_{F} - (\varphi_{f}^{2} + \varphi_{F}^{2} )/2$, and the average effects of alleles are given by $\alpha_{\gamma } = a_{\gamma } + (p_{G} - p_{g} )d_{\gamma }$ and $\alpha_{\varphi } = a_{\varphi } + (p_{F} - p_{f} )d_{\varphi }$, where *p* denotes allele frequency [15]. Hence, in Eqs. 7 and 11 of [5], $\gamma_{g} - \gamma_{G}$ should be replaced by $\alpha_{\gamma }$, and $\varphi_{f} - \varphi_{F}$ should be replaced by $\alpha_{\varphi }$. For example, for $\gamma_{g} = 1$ and $\gamma_{G} = 0.6$, genotypic values are $\gamma_{gg} = 1$, $\gamma_{gG} = 0.6$ and $\gamma_{GG} = 0.36$, the additive effect is $a_{\gamma } = (1 - 0.36)/2 = 0.32$, the dominance deviation is $d_{\gamma } = 0.6 - (1 + 0.36)/2 = - 0.08$, and the average effect is $\alpha_{\gamma } = 0.32 - 0.08\,(p_{G} - p_{g} )$.

In this study, we assumed a model with two bi-allelic loci, i.e. one locus that affects individual susceptibility and one locus that affects individual infectivity. Furthermore, we assumed that which locus affects infectivity and which locus affects susceptibility, are known. This may be the case with candidate gene approaches which include only the genes for which the function is related to the trait of interest. The effect of the putative causative gene is then examined by association study. In such studies, the GLM developed here can be applied to estimate and confirm the effect of the candidate gene on the trait of interest. However, applying a candidate gene approach is limited because it relies on knowing the functional relation between the genes and the trait of interest. The recent advances in molecular genomics allow us to genotype individuals for thousands of SNPs, and to perform GWAS in which all SNPs are examined for their association with the trait of interest. The GLM developed here can also be used in GWAS that aim at identifying genes associated with susceptibility and/or infectivity. In such studies, it is not known whether a SNP affects infectivity and/or susceptibility. Hence, this has to be inferred from the significance of the estimated effects. To avoid the need to test all combinations of two SNPs, one could first screen SNPs for susceptibility effects, and then fit only the significant loci for susceptibility effects, together with all other loci for infectivity effects. Moreover, when modified so that gene effects are estimated as random effects, our model can probably be used for polygenic traits, for example in genomic prediction, for which effects of all genes are estimated simultaneously and the interest lies in predicting the breeding value of entire genotypes [18].

## Conclusions

We have developed a generalized linear model to estimate the relative effects of genes on individual susceptibility and infectivity. This model may be used in genome-wide association studies that aim at identifying genes that are involved in the prevalence of infectious diseases.

## Figures and Tables

**Fig. 6:**
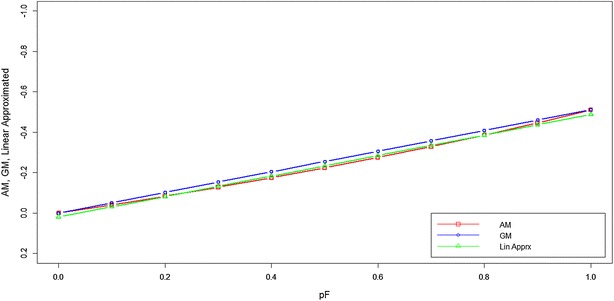
Arithmetic mean (AM), geometric mean (GM) and linear approximation of best fitted line. *Each* *line* is a function of allele frequency, $p_{F}$, with input values for $\varphi_{f}$ and $\varphi_{F}$ being 1 and 0.6, respectively

## Appendix

### Geometric mean versus arithmetic mean in the estimation of gene effects on infectivity

In this Appendix, we address one issue regarding the quality of the two estimators, which we use to recover the genetic parameters. In general, one would like to have estimators that give consistent estimates of the parameters. This implies that both the variance and the bias of the estimators for a sufficiently large dataset (size n) can be brought arbitrarily close to zero. Expressed in formulas for the relative infectivity and the relative susceptibility, which are the two parameters that we want to estimate, these requirements look like this:

$$\mathop {\lim }\limits_{n \to \infty } \widehat{{\left( {\frac{{\varphi_{F} }}{{\varphi_{f} }}} \right)}} - \left( {\frac{{\varphi_{F} }}{{\varphi_{f} }}} \right) = 0,$$

$$\mathop {\lim }\limits_{n \to \infty } \widehat{{\left( {\frac{{\gamma_{G} }}{{\gamma_{g} }}} \right)}} - \left( {\frac{{\gamma_{G} }}{{\gamma_{g} }}} \right) = 0,$$

$$\mathop {\lim }\limits_{n \to \infty } {\text{var}}\widehat{{\left( {\frac{{\varphi_{F} }}{{\varphi_{f} }}} \right)}} = 0,$$

$$\mathop {\lim }\limits_{n \to \infty } {\text{var}}\widehat{{\left( {\frac{{\gamma_{G} }}{{\gamma_{g} }}} \right)}} = 0.$$

In addition, one would like to know how fast the estimators approach these limits. That analysis is presented in the main text and is done by comparing simulations to the true values. There is, however, an issue with the asymptotic unbiasedness of the effect on infectivity (the first equation): the estimator for the effect of the relative infectivity is not unbiased, but instead we will show below that:

$$\mathop {\lim }\limits_{n \to \infty } \frac{{\widehat{{Log\left( {\frac{{\varphi_{F} }}{{\varphi_{f} }}} \right)}}}}{{Log\left( {\frac{{\varphi_{F} }}{{\varphi_{f} }}} \right)}} = m\left( {\frac{{\varphi_{F} }}{{\varphi_{f} }}} \right),$$

and we will derive the expression for the function m(.) and will show that it is close to 1 and always smaller or equal to 1. Note that m(.) = 1 means no bias and m(.) < 1 means underestimation of the effect.

As explained in the main text, the transmission rate parameter (*β*) is the product of the contact rate (c), susceptibility (γ) and infectivity ($\varphi$). Applying the complementary log–log link function results in Log(*β*) being in the expression for the expected value of the dependent variable. Thus, to see whether a linear relation is obtained between the explanatory variables to explain the expected value of the dependent variable, we can write that:

$${\rm Log}(\beta) = {\rm Log}(c) + {\rm Log}(\gamma ) + {\rm Log}(\varphi ).$$

The heterogeneity in Log(γ) is straightforwardly incorporated in the model since each recipient counted in the dependent variable is only one type of susceptible individual. Thus, take γ_g_ = 1 and the other type has γ_*G*_, then:

$$Log(\beta ) = Log(c) + Index_{G} Log(\gamma_{G} ) + Log(\varphi ),$$

where $Index_{G}$ is equal to 1 if the recipient is G and 0 when the recipient is of type g, with additional modification for the three genotypes as explained in the main text. Thus, the estimated parameter is asymptotically unbiased using the GLM method.

For heterogeneity in $\varphi$, it is not straightforward because we are dealing with the arithmetic mean ($\varphi_{AM}$) across all types of infectious individuals in the populations as was derived in the main text. Let us again look at the case with only two types of infectious individuals:

$$Log(\beta ) = Log(c) + Index_{G} Log(\gamma_{G} ) + Log(\varphi_{F} p_{F} + \varphi_{f} p_{f} ),$$

where $p_{F}$ is the explanatory variable $(p_{f} = 1 - p_{F} ).$

In order to obtain linearity in the explanatory variable for infectivity, $\varphi_{AM}$ is replaced by geometric mean $\varphi_{GM}$ with $\varphi_{GM} = \prod\nolimits_{j = 1}^{n} {\varphi_{j}^{{p_{j} }} }$. The equation with two types of infectious individuals becomes:

$$Log(\beta ) = Log(c) + Index_{G} Log(\gamma_{G} ) + p_{F} Log(\varphi_{F} ) + p_{f} Log(\varphi_{f} ).$$

This is a linear equation in $p_{F}$, the explanatory variable, because $p_{f} = 1 - p_{F}$.

Now, we calculate the systematic error (bias) made by the approximation of the arithmetic mean ($\varphi_{AM}$) by a geometric mean ($\varphi_{GM} )$). For a bi-allelic genetic model, where there are two alleles, i.e. $\varphi_{F}$ and $\varphi_{f}$, with a frequency $p_{F}$ and $(1 - p_{F} )$, respectively, the ${\text{Log(}}\varphi_{AM} )$ expression for the two alleles can be written as:

$${\text{Log}}(\varphi_{AM} ) = {\text{Log}}(p_{F} \varphi_{F} + (1 - p_{F} )\varphi_{f} ),$$

A1 $${\text{Log}}(\varphi_{AM} ) = {\text{Log}}((\varphi_{F} - \varphi_{f} )p_{F} + \varphi_{f} ).$$

Thus, the effect of $\varphi_{F}$ compared to $\varphi_{f}$ is measured by the coefficient of $p_{F}$, i.e., the slope of the linear expression within the logarithm, but this is not a linear model. Note that if the number of data points (n) becomes larger and larger, the expected (average) observed values, i.e. the number of cases (y) among the number of susceptible (S), will after applying the cloglog link function become arbitrary close to the expression A1, or:

$$\mathop {\lim }\limits_{n \to \infty } cloglog\left( {E\frac{y}{S}} \right) = C_{0} + {\text{Log}}((\varphi_{F} - \varphi_{f} )p_{F} + \varphi_{f} ).$$

To obtain a linear model, we take the ${\text{Log}}(\varphi_{GM} )$ expression for the two alleles which can be written as:

$${\text{Log}}(\varphi_{GM} ) = {\text{Log}}(\varphi_{F}^{{p_{F} }} \varphi_{f}^{{1 - p_{F} }} ),$$

$${\text{Log}}(\varphi_{GM} ) = p_{F} {\text{Log(}}\varphi_{F} )+ (1 - p_{F} ){\text{Log(}}\varphi_{f} ) ,$$

A2 $${\text{Log}}(\varphi_{GM} ) = p_{F} {\text{Log}}\left( {\frac{{\varphi_{F} }}{{\varphi_{f} }}} \right) + {\text{Log(}}\varphi_{f} ).$$

Now the effect of the allele $\varphi_{F}$ compared to $\varphi_{f}$ is measured by the ratio of the two values instead of the difference as in expression A1. This ratio can thus be calculated as the antilog of the regression coefficient of $p_{F}$ which is the explanatory variable. In other words, from the GLM in Eq. (8) in this paper, the estimated Log of the ratio of $\varphi_{F}$ over $\varphi_{f}$ is obtained from the regression coefficient $c_{2}$.

Now the next issue that we address in this Appendix is to fit a linear equation for the ${\text{Log}}(\varphi )$ as a function of allele frequency $(p_{F} )$ which is:

A3 $$\log (\varphi_{LIN} ) = A \cdot p_{F} + B.$$

If, in fact, the transmission depended on the $\varphi_{GM}$ rather than on $\varphi_{AM}$, we would have:

$$A = \log \left( {\frac{{\varphi_{F} }}{{\varphi_{f} }}} \right)\quad{\text{and }}\quad B = \log (\varphi_{f} ).$$

However, since the data come from a process where the observed $\varphi$ is in fact the $\varphi_{AM}$, the resulting linear relationship will not (necessarily) have $A = \log \left( {\frac{{\varphi_{F} }}{{\varphi_{f} }}} \right)$.

As we are interested in the allele effects, we need to estimate the slope of the line, i.e. the regression coefficient (A) of $p_{F}$, and compare it to $\log \left( {\frac{{\varphi_{F} }}{{\varphi_{f} }}} \right)$. To determine A, we need to find the best fitting linear relationship for ${\text{Log}}(\varphi_{LIN} )$ from ${\text{Log}}(\varphi_{AM} )$ data (Fig. 6). This was done and we showed that this estimated A is very close to ${\text{Log}}\left( {\frac{{\varphi_{F} }}{{\varphi_{f} }}} \right)$, and we were able give an explicit expression for the bias with respect to this true value.

### Derivation of the expression for fitted line through Log($\varphi_{AM}$)

The following shows how the A and B for the linear model in Eq. (A3) can be obtained when this linear model is fitted to data generated by the non-linear relation between the explanatory variable ($p_{F}$) and the observed effect ${\text{Log}}(\varphi_{AM} )$. For each value of $p_{F}$, we observe a corresponding value for ${\text{Log}}(\varphi_{AM} )$, which gives a nonlinear relationship [Eq. (A1); Fig. 6]. Thus, in order to obtain a linear relationship between the parameter of interest $(p_{F} )$ and the dependent variable, we fit a line through this nonlinear relationship from which we estimate the effect (in this case, the Log of the effect of $\varphi_{F}$ compared to $\varphi_{f}$). To fit a line through the true relationship ${\text{Log}}(\varphi_{AM} )$, we sample random values for $p_{F}$ from a uniform distribution from 0 to 1 and calculate corresponding values of ${\text{Log}}(\varphi_{AM} )$ from Eq. (A1). If we draw a least squares regression line through the random numbers drawn from these [$p_{F}$, ${\text{Log}}(\varphi_{AM} )$] pairs, the line passes through the average values sampled: $\overline{p}_{F}$ and $\overline{{{\text{Log}}(\varphi_{AM} )}}$.

This allows us to find *B*, since we know that $\overline{p}_{F} = {1/2}$, and $\overline{{{\text{Log}}(\varphi_{AM} )}}$ is:

$$\overline{{{\text{Log}}(\varphi_{AM} )}} = \mathop \int \limits_{0}^{1} \log \left[ {(\varphi_{F} - \varphi_{f} )p_{F} + \varphi_{f} } \right]dp_{F} ,$$

$$\overline{{{\text{Log}}(\varphi_{AM} )}} = \frac{{(\varphi_{f} - \varphi_{F} ) + \varphi_{F} \log \varphi_{F} - \varphi_{f} \log \varphi_{f} }}{{\varphi_{F} - \varphi_{f} }}.$$

Hence, since $\overline{{{\text{Log}}(\varphi_{AM} )}} = A \cdot \overline{p}_{F} + B$, and $\overline{p}_{F} = {1/2}$,

$$B = \overline{{{\text{Log}}(\varphi_{AM} )}} - \frac{1}{2} A$$, and thus

$${\text{Log}}(\varphi_{LIN} ) = A \cdot p_{F} + \overline{{{\text{Log}}(\varphi_{AM} )}} - \frac{1}{2} \cdot A.$$

Now we have an equation with only one unknown (A) and the solution for A, denoted *A*_*min*_, can be found by taking the least squares optimization. This means that we can find the minimum solution for the squared difference between the ${\text{Log}}(\varphi_{AM} )$ and Log$(\varphi_{LIN} )$ derived above.

$$A_{min} = MIN_{A} \mathop \int \limits_{0}^{1} \left( {A \cdot p_{F} + \overline{{\log (\varphi_{AM} )}} - \frac{1}{2} \cdot A - {\text{Log(}}(\varphi_{F} - \varphi_{f} ) p_{F} + \varphi_{f} ) } \right)^{2} d p_{F} .$$

This integral was evaluated with symbolic computer algebra using Mathematica. This is a straightforward evaluation but, at first, the expressions appear to be very big. Thus, we undertook some simplifications to find the A for which the minimum of the expression is attained. As the part between brackets is a linear expression in A, the result of the above equation is a quadratic equation in A, and thus can be written as:

$$MIN_{A} (K_{2} A^{2} + K_{1} A + K_{0} ).$$

The equation between brackets is for an upward open parabola (if $K_{2} > 0$) and the minimum of this parabola is attained for:

$$A_{min} = \frac{{ - K_{1} }}{{2K_{2} }}$$, where (when $$\varphi_{F} \ne \varphi_{f} ){:}$$

$$K_{1} = \frac{{ - 6 \varphi_{F} \left( {\varphi_{F} - \varphi_{f} } \right) - 6\left( {\varphi_{F} - \varphi_{f} } \right)\varphi_{f} + 12\varphi_{F} \varphi_{f} {\text{Log}}(\frac{{\varphi_{F} }}{{\varphi_{f} }})}}{{12(\varphi_{F} - \varphi_{f} )^{2} }} = \frac{{ - \varphi_{F}^{2} + \varphi_{f}^{2} + 2\varphi_{F} \varphi_{f} {\text{Log}}\left( {\frac{{\varphi_{F} }}{{\varphi_{f} }}} \right)}}{{2\left( {\varphi_{F} - \varphi_{f} } \right)^{2} }}$$

And

$$K_{2} = \frac{{\varphi_{f} (\varphi_{f} - \varphi_{F} ) + \varphi_{F} (\varphi_{F} - \varphi_{f} )}}{{12(\varphi_{f} - \varphi_{F} )^{2} }} = \frac{1}{12},$$

thus

$$A_{min} = \frac{{3\varphi_{F}^{2} - 3\varphi_{f}^{2} - 6\varphi_{F} \varphi_{f} {\text{Log}}\left[ {\frac{{\varphi_{F} }}{{\varphi_{f} }}} \right]}}{{(\varphi_{f} - \varphi_{F} )^{2} }}.$$

Then, both the numerator and denominator of the above equation were divided by $\varphi_{f}^{2}$ and this resulted in:

$$A_{min} = \frac{{3\left( {\frac{{\varphi_{F} }}{{\varphi_{f} }}} \right)^{2} - 3 - 6\left( {\frac{{\varphi_{F} }}{{\varphi_{f} }}} \right){\text{Log}}\left[ {\frac{{\varphi_{F} }}{{\varphi_{f} }}} \right]}}{{(1 - \left( {\frac{{\varphi_{F} }}{{\varphi_{f} }}} \right))^{2} }}.$$

Thus, *A*_*min*_ is a function of $\frac{{\varphi_{F} }}{{\varphi_{f} }}$ only. Note the similarity with Eq. (A2) where $A = \log \left( {\frac{{\varphi_{F} }}{{\varphi_{f} }}} \right)$. It should be noted that the A_min_ is the estimate (C_2_) that will be obtained asymptotically (i.e. when n → ∞) from the GLM in Eq. (8). Thus, we investigated the relation of this estimated value to the true expected value ${\text{Log}}\left[ {\frac{{\varphi_{F} }}{{\varphi_{f} }}} \right]$, to quantify the bias due to our approach.

Let us assume that $\frac{{\varphi_{F} }}{{\varphi_{f} }} = x$, thus the above equation can be simplified as:

$$A_{min} = \frac{{3(x^{2} - 1 - 2x{\text{Log}}(x))}}{{(1 - x)^{2} }}.$$

Still, $A_{min} \ne \log \left( {\frac{{\varphi_{F} }}{{\varphi_{f} }}} \right) \ne \log (x)$, hence there is a non-zero bias. However, we can now define m(x) by $A_{min} = m(x) \cdot \log (x)$. The value of *m* quantifies the amount of relative bias that is obtained as a result of the geometric approximation; a value *m* = 1 indicates a zero bias. Thus:

A4 $$m(x) = \frac{{A_{min} }}{{{\text{Log}}(x)}} = \frac{{3(x^{2} - 1 - 2x{\text{Log}}(x))}}{{(x - 1)^{2} {\text{Log}}(x)}}.$$

Equation (A4) quantifies the amount of bias, the magnitude of which is numerically investigated below. However, first it is necessary to check Eq. (A4) using some relationships that are known to hold for the underlying problem, for example:

$m(x) = m\left( {\frac{1}{x}} \right)$, since it should not matter which allele is coded *F* or *f*.

$\mathop {\lim }\nolimits_{x \to 1} m(x) = 1$, since the arithmetic and geometric mean are identical when $\varphi_{F} = \varphi_{f}$.

$\mathop {\lim }\nolimits_{x \to 0} m(x) = 0$, and $\mathop {\lim }\nolimits_{x \to \infty } m(x) = 0$, since we always underestimate the effect because 0 ≤ *m*(*x*) ≤ 1 and thus it seems that *m*(*x*) will have to approach zero when the real effect becomes infinitely large (i.e., either *x* = 0 or *x* → ∞). As a result, we will estimate a finite value for the effect even when the effect is infinite and, thus, we make an infinitely large error, i.e. *m*(*x*) = 0. All conditions hold as it can be checked using Eq. (A4).

Going back to our paper, we now look at Eq. (8), which is,

$${\text{cloglog}} E\left[ {\frac{{y_{i} }}{{n_{i} }}} \right] = c_{0} + c_{1} index_{G,i} + c_{2} Num_{F} + log\left( {\frac{I}{N}} \right),$$

where $c_{2}$, is the regression coefficient that we estimate. In other words, when applying the geometric mean approximation, we assume $Est(\log (x)) = \widehat{c}_{2}$, whereas in fact, ${\text{Log}}(x) = \frac{{\widehat{c}_{2} }}{m(x)}$, when we correct for the geometric mean approximation.

Since we assumed that $\varphi_{f} = 1$, then ${\text{Log}}(x) = {\text{Log}}\left( {\frac{{\varphi_{F} }}{{\varphi_{f} }}} \right) = {\text{Log}}(\varphi_{F} )$. Thus:

A5 $${\text{Log}}(\varphi_{F} ) = \frac{{\widehat{c}_{2} }}{{m(\varphi_{F} )}},$$

where

A6 $$m(\varphi_{F} ) = \frac{{3((\varphi_{F} )^{2} - 1 - 2(\varphi_{F} ){\text{Log[}}\varphi_{F} ])}}{{((\varphi_{F} ) - 1)^{2} {\text{Log}}[\varphi_{F} ]}}.$$

The result from Eq. (A6) quantifies the amount of error that was obtained as a result of the geometric approximation. An $m(\varphi_{F} ) = 1$ indicates no error, an $m(\varphi_{F} ) < 1$ indicates underestimation, while an $m(\varphi_{F} ) > 1$ indicates overestimation of $\varphi_{F}$. As 0 < $m(\varphi_{F} )$ < 1, the estimated value is always too small. Hence, the geometric mean approximation is conservative. Furthermore, ${\text{m}}(\varphi_{F} ) = m\left( {\frac{1}{\varphi }_{F} } \right)$ for all $\varphi_{F}$ and m(1) = 1, the further $\varphi_{F}$ is away from 1 (the larger effect), the higher the error. Roughly speaking for values of $\varphi_{F}$ between 0.333 and 3, the error is smaller than 5 %; i.e., 0.95 < *m* < 1.

Now that we have quantified the amount of bias [Eqs. (A5) and (A6)], we can obtain the correct value. Note that in Eq. (A5), the (true) value of $\varphi_{F}$ appears on both sides of the equation. Thus, we need an iterative procedure to obtain the real value. First, $\widehat{\varphi }_{F}$ is calculated by taking the exponential of $c_{2}$ from the GLM analysis. Then, the error $m(\widehat{\varphi }_{F} )$ [Eq. (A6)] followed by the new value for in $\log (\varphi_{F} )$ in Eq. (A5) are estimated. $\widehat{\varphi }_{F}$ is then again calculated by taking the exponential of $\log (\varphi_{F} )$. This iteration process is then allowed to continue until there is no change in $\widehat{\varphi }_{F}$.

In the tables below, the biases obtained as a result of the geometric mean approximation are presented for the different scenarios investigated in the main text. This bias is calculated as the difference between $\widehat{\varphi }_{F}$ that is obtained after accounting for the error as a result of geometric mean approximation and $\widehat{\varphi }_{F}$ that is obtained when the error is not accounted for. Note that there is additional bias with respect to the true value which is of course known from the simulations. This bias is also small but larger than asymptotically expected from the GM approximation.

See Tables 3, 4, 5 and Fig. 6.

**Table 3:** Biases in estimated $\varphi_{F}$ for scenario 1

*r* = 0	*r* = 0.25	*r* = 0.5625	*r* = 1
0.000674	0.002126	0.002569	0.002698

**Table 4:** Biases in estimated $\varphi_{F}$ for scenario 2

	*r* = 0	*r* = 0.25	*r* = 0.5625	*r* = 1
*R*_*0*_ = 0.6	0.000452	0.001309	0.002122	0.002706
*R*_*0*_ = 1.2	0.000674	0.002126	0.002569	0.002698
*R*_*0*_ = 1.8	0.001889	0.002498	0.002612	0.002671
*R*_*0*_ = 2.5	0.002774	0.002467	0.002531	0.002565
*R*_*0*_ = 3.1	0.003111	0.002364	0.002364	0.002492
*R*_*0*_ = 3.7	0.003425	0.002181	0.002201	0.002375
*R*_*0*_ = 4.3	0.003891	0.002032	0.002062	0.002223
*R*_*0*_ = 4.9	0.004184	0.001891	0.001866	0.00208
*R*_*0*_ = 5.5	0.004707	0.001798	0.001705	0.001903
*R*_*0*_ = 6.1	0.005194	0.00167	0.001572	0.001743

**Table 5:** Biases in estimated $\varphi_{F}$ for scenario 3

	*r* = 0	*r* = 0.25	*r* = 0.5625	*r* = 1
Small difference	8.60436E−05	9.26451E−05	7.50199E−05	5.75224E−05
Moderate difference	0.000674	0.002126	0.002569	0.002698
Large difference	0.002963	0.013645	0.017404	0.018417
